# The effect of antipsychotic medication and the associated hyperprolactinemia as a risk factor for periodontal diseases in schizophrenic patients: a cohort retrospective study

**DOI:** 10.1186/s12903-023-03404-1

**Published:** 2023-10-24

**Authors:** Rania Shalaby, Ahmed Elmahdy, Christine Mikhail

**Affiliations:** 1 Department of Oral Medicine, Diagnosis and Periodontology, Faculty of Dentistry, Fayoum University, Fayoum, Egypt; 2 Faculty of Medicine, MUST University, Giza, Egypt

**Keywords:** Antipsychotics, Hyperprolactinemia, Early prevention, Periodontal disease, Schizophrenia

## Abstract

**Background:**

Periodontal disease is a major health problem that results in tooth loss and thus affects oral health, which affects quality of life. In particular, schizophrenic patients are at higher risk for periodontal disease due to several factors, including the effect of antipsychotic medications received by those patients. Accordingly, the aim of the present cohort retrospective study is to explore the effect of antipsychotics on periodontal health and the possible effect of antipsychotic-induced hyperprolactinemia as a risk factor for periodontal disease progression in schizophrenic patients.

**Methods and outcomes:**

The study population consisted of three groups: Group A (n = 21): schizophrenic patients that have been taking “prolactin-inducing” antipsychotics for at least 1 year; Group B (n = 21): schizophrenic patients who have been taking “prolactin-sparing” antipsychotics for at least 1 year; and Group C (n = 22): newly diagnosed schizophrenic patients and/or patients who did not receive any psychiatric treatment for at least 1 year. The study groups underwent assessment of periodontal conditions in terms of pocket depth (PD), clinical attachment loss (CAL), gingival recession, tooth mobility, and bleeding on probing (BOP). Also, bone mineral density was evaluated using DEXA scans, and the serum prolactin level was measured by automated immunoassay.

**Results:**

Results revealed a statistically significant difference in PD, CAL, and serum prolactin levels (P ≤ 0.001, P = 0.001, and P ≤ 0.001, respectively) among the 3 study groups. For both PD and CAL measurements, group A has shown significantly higher values than both groups B and C, whereas there was no statistically significant difference between the values of groups C and B. Concerning serum prolactin levels, group A had significantly higher values than groups B and C (P ≤ 0.001 and P ≤ 0.001 respectively). There was a statistically significant difference (P ≤ 0.001) between the 3 study groups in terms of bone mineral density. Moreover, there was a statistically significant direct relation between serum prolactin level and other parameters including clinical attachment loss, pocket depth measurements and bone mineral density.

**Conclusion:**

According to our results, it could be concluded that all antipsychotics contribute to the progression of periodontal disease, with a higher risk for prolactin-inducing antipsychotics. However, further long term, large sampled, interventional and controlled studies are required to reach definitive guidelines to allow clinicians properly manage this group of patients.

## Introduction

Periodontal disease is a microbial, chronic inflammatory disease that occurs in susceptible individuals. Primarily, it is due to the interaction of two forces: microbial imbalance and the host immune response [1, 2]. It is clinically identified as a clinical loss of attachment (CAL) by circumferential evaluation of the erupted teeth with a standard periodontal probe in relation to the cement-enamel junction (CEJ) [3]. It results in pocket depth (PD) formation, gingival recession, and bone loss [4].

Periodontitis is considered the sixth most prevalent disease and affects approximately 10% of the adult population worldwide [5, 6]. It constitutes a serious socioeconomic and public health issue as it results in the destruction of the tooth attachment apparatus and eventual tooth loss. Evidence from research indicates that approximately 20% of tooth loss is caused by periodontitis [7]. Consequently, esthetics and mastication are compromised, which affects the quality of life [8–11]. Due to the high prevalence of periodontal disease as well as its great impact on socioeconomic aspects and quality of life, the selection of the most suitable treatment modality is mandatory. It is worth noting that the definition of health, as claimed by the World Health Organization (WHO), integrate both psychological and physical functioning [12].

Principally, three main therapeutic categories can be established, including; periodontally sound, gingivitis, and periodontitis. In the periodontitis category, the fundamental treatment strategies for chronic and aggressive periodontitis cases are entirely distinct. Basically, comprehensive periodontal therapy is divided into four phases: phase I, initial or cause-related therapy, phase II, surgical therapy, phase III, periodontal reconstruction, and phase IV, periodontal maintenance. For gingivitis and chronic periodontitis, the classic non-surgical and surgical techniques can cure inflammation, halt the progression of periodontal destruction, and restore gingival-periodontal health, and the outcome is quite predictable. Long-term outcomes are contingent on patient motivation and maintenance [13].

Based on the WHO concept of health, periodontal treatment outcome should not be simply evaluated on the basis of periodontal parameters (e.g.: CAL) but, the overall patient’s experience and quality of life should be considered. In that sense, deep understanding of the etiology and pathogenesis seems essential to identify the most adequate treatment option [12].

Traditionally, the etiopathogenesis of periodontal disease involves abstinence from oral hygiene, leading to plaque accumulation, gingival inflammation, and the development of clinical gingivitis. Over time, with augmentation of microbial burden in dental plaque and proximity of subgingival plaque to the pocket epithelium, induction of a cellular inflammatory response in connective tissue occurs, leading to progression into periodontitis. Over the years, there has been a paradigm shift in the understanding of the etiopathogenesis of periodontitis. In other words, although the microbial load that resides in dental plaque represents the principal causative agent, the initiation of the disease appears to be a more sophisticated process that requires the interplay of several risk factors [14].

Among the well-recognized risk factors, genetics and lifestyle play a crucial role in the development and progression of periodontal disease. For instance, poor oral hygiene, obesity, metabolic syndrome, osteoporosis, a lack of dietary calcium and vitamin D, diabetes mellitus (DM), cardiovascular diseases (CVS), psychomotor function disorders, smoking, and AIDS have been identified as possible risk factors [15–18]. In this context, the existence of a bidirectional relationship between systemic conditions and periodontitis has been widely accepted. In part, systemic disease could aggravate periodontal disease through augmentation of microbial burden and inflammatory response, which reciprocally synergizes with periodontal pathogens and the locally activated lymphocyte with spillover of inflammatory cytokines that spread out via circulation into extra-oral tissues [19–24].

In addition, some medications, such as antihypertensive, anticholinergic, and antipsychotic drugs, were implicated in the aggravation of periodontal disease. This effect has been mainly attributed to the hyposalivation induced by these drugs. For instance, quetiapine, olanzapine, and risperidone may induce xerostomia, which in turn affects oral health [25, 26].

To date, the correlation between antipsychotics and periodontal diseases has not been extensively studied. Only a few studies have addressed the link between the use of antipsychotics and periodontal conditions in schizophrenia patients [27–29].

Schizophrenic patients are at high risk of periodontal diseases due to the interplay of several risk factors, including neglecting oral hygiene, smoking, and the prescribed antipsychotic medications. Since schizophrenia is a chronic disease, treatment with antipsychotics may be prolonged, imposing further compromise on periodontal health [30–33]. Roughly 10% of the global population suffers from a mental condition at any one time [10]. The underlying cause of schizophrenia, one of the most common psychotic disorders, is the disconnection between thoughts and emotions [34]. Negative symptoms include anhedonia and apathy; positive symptoms include hallucinations and delusions, as well as difficulties with communication and coordination [27].

Classically, antipsychotic medications have been categorized based on their clinical use. Primarily, these drugs have been clinically used for schizophrenia and related psychoses. Although they share a common dopaminergic mechanism of action (dopamine D2 receptor antagonism), they show variability regarding clinical efficacy and tolerability [35–38]. They are mainly classified into two groups: the first-generation antipsychotics (FGAs), or typical, and the second-generation antipsychotics (SGAs), or atypical. FGAs such as chlorpromazine, thioridazine, perphenazine, fluphenazine, and haloperidol act by blocking dopamine (D2 receptors), and they are further classified into low- or high-potency FGAs according to their capacity to bind D2 receptors. The SGAs, including drugs like Clozapine, Aripripazole, Quetiapine, Olanzapine, and Risperidone, are weaker D2 receptor blockers than FGAs, with high affinity for blocking serotonin (5-HT2) receptors, alpha (α) adrenergic receptors, and H1 receptors, hence the term “Dopamine-Serotonin Antagonists.“ [39, 40].

The majority of antipsychotics have side effects like CVS and blood dyscarias, as well as extrapyramidal symptoms like dystonia, akathisia, tardive dyskinesia, Pseudo-parkinsonism, and neuroleptic malignant syndrome. Adrenergic blockade effect causes orthostatic hypotension and tachycardia, while the anticholinergic effect causes xerostomia, constipation, and blurred vision. Also, the patient may experience mental confusion, lethargy, or drowsiness [39].

The American Psychiatric Association claims that despite the metabolic side effects of SGAs, these medications are the first-line treatment for schizophrenia because they cause fewer extrapyramidal symptoms than FGAs. Aside from clozapine, which causes sialorrhea, the majority of antipsychotics result in diminished salivary flow, thereby reducing the purifying and protective effects of saliva, which accelerates the progression of periodontal diseases [28].

Currently, there is no clear guidance for clinicians that aids in the proper selection of the most suitable antipsychotic agent [35]. In aggregate, most antipsychotics cause hyperprolactinemia. Meanwhile, only limited data are available on the effect of antipsychotic medications on physical and oral health. Nevertheless, endocrinal and immunological changes, as well as growth hormone alteration, have been commonly reported with hyperprolactinemia [41].

Notably, antipsychotic-induced hyperprolactinemia is the possible mechanism underlying antipsychotic-induced osteoporosis; however, some of the SGAs, such as olanzapine, quetiapine, and aripeprazole, have a minimal effect on serum prolactin levels [42]. Although the World Health Organization (WHO) does not list antipsychotics as an osteoporosis-inducing agent, it is hypothesized that they may influence bone turnover by augmenting hyperprolactinemia. However, a clear association has not been established yet [43, 44].

Interestingly, it has been postulated that antipsychotic-induced hyperprolactinemia could be one possible risk factor for the progression of periodontal disease due to the increased propensity for alveolar bone resorption [45]. Accordingly, we employed the present study to identify the effect of some of the most commonly prescribed antipsychotics indicated for schizophrenia on periodontal health. Furthermore, we investigated the possible contribution of antipsychotic-induced hyperprolactinemia as a risk factor for periodontal disease progression in schizophrenic patients.

## Methods

### Study design

The current study is a cohort retrospective study that was performed on schizophrenic patients.

### Study setting

The study was carried out over a period of four months from the first of September 2022 until the first of January 2023. All subjects were recruited from the outpatient clinic of the Department of Psychiatry, Faculty of Medicine, MUST University.

The patients’ database from the department of psychiatry was filtered, and all patients with the diagnosis of schizophrenia [46] and fulfilling eligibility criteria were contacted. Those who agreed to be enrolled in the study received a thorough explanation of the study’s purpose and potential benefits, with an emphasis on maintaining their confidentiality. Each participant completed an informed consent form prior to the start of the study.

For patients previously diagnosed with schizophrenia, medication data was acquired from prescription files, including the type and duration of the prescribed antipsychotic or other medications. For newly diagnosed patients, psychiatric assessment and recording of demographic variables such as age, gender, smoking habits, systemic medical condition, duration of psychiatric disease, and type and duration of antipsychotic medication were performed by the second investigator (EM.A.). Then, all subjects were referred, with their records and files, to the Faculty of Dentistry at ACU and Fayoum University for periodontal assessment and sample collection.

The research has been registered and exempted by Institutional Review Board Organization IORG0010868, Faculty of Oral & Dental Medicine, AL Ahram Canadian University. Research Number: IRB00012891 #18.

### Eligibility criteria

The included patients are those: (1) with the diagnosis of schizophrenia [46]; (2) diagnosed with chronic periodontitis [3]; (3) over 20 years old; and (4) with at least 20 remaining teeth. To avoid potential confounding factors, the excluded patients are: (1) those with systemic conditions that may affect periodontal status, such as DM, CVS, metabolic syndrome, osteoporosis, AIDS, and chronic alcoholism [15, 16, 29]; (2) those with local factors that may aggravate and predispose for periodontal diseases, such as orthodontic and prosthetic appliances and parafunctional habits [16, 47–49]; (3) those receiving any systemic medication and/or systemic antibiotics for the past 6 months; (4) Patients undergoing any type of periodontal treatment for the past year; (5) patients within the childhood and adolescent psychiatry section; and (6) patients who received antipsychotic medication for ≥ 12 months (7) Localized periodontitis in which ≤ 30% of teeth are involved.

### Estimating the sample size

The sample size was calculated considering a type I error (α) of 0.05 and a power (1-) of 0.9. Based on a previous study by Djamaluddin et al. [28] that used proportions, inequality, and two independent groups (Fisher’s exact test) to compare patients with PD of 4 mm to those with no PD identified in patients receiving antipsychotics for 12 months, the sample size was calculated and found to be a total of 64 patients. The application G*Power 3.1.9.7 [16] was used to determine the sample size needed for the study.

### Participants

The study population was divided into three groups: Exposure groups were divided according to their “prolactin-inducing” or “prolactin-sparing” effect [41, 50–59] into:

#### Group A (n = 21)

schizophrenic patients that have been taking antipsychotic medication that may induce hyperprolactinemia (FGAs and SGAs; amisulpride, risperidone, and paliperidone) for at least 1 year.

#### Group B (n = 21)

schizophrenic patients who have been taking antipsychotics that do not have a significant effect on serum prolactin levels (in SGAs, clozapine, quetiapine, olanzapine, ziprasidone, and aripiprazole) for at least 1 year.

#### Group C (n = 22)

newly diagnosed schizophrenic patients and/or patients who did not receive any psychiatric treatment for at least 1 year.

### Outcome measurements

The primary outcome was the assessment of periodontal condition in all study groups measured in terms of CAL, PD, tooth mobility, gingival recession, and bleeding on probing (BOP), while the secondary outcomes were the evaluation of bone mineral density (BMD) and the serum prolactin level (measured by an automated immunoassay in ng/ml).

### Assessment of clinical data and patient condition

Assessment of mental health: The Positive and Negative Syndrome Scale (PNSS) [46] was used to evaluate each patient’s clinical history and current mental health state.

#### Periodontal evaluation

All teeth were evaluated and recorded. The means for the following parameters were computed: PD, CAL, and BOP [60, 61]. Using a manual periodontal probe (Williams’ periodontal probe, PCP-12; Hu-Friedy, Chicago, IL, USA), PD and CAL measurements were collected on six surfaces per tooth (mesio-buccal, mid-buccal, disto-buccal, and mesio-lingual, mid-lingual, disto-lingual, or palatal surface). While CAL measures the distance between the cement-enamel junction of the tooth and the deepest aspect of the pocket, PD measures the distance between the gingival margin and the deepest part of the pocket. The total mean PD of the six locations for each tooth was computed for each patient, and the distance was recorded to the nearest millimeter [62]. Sulcus depths between 0 and 2 mm were regarded as normal [57]. Gingival recession was measured from the CEJ to the marginal border of the soft tissue on the buccal and lingual sides of each tooth. Gingival recession, if present, was only used to calculate CAL by its addition to PD.

Four surfaces per tooth were examined for BOP readings: the mesial, distal, buccal, and lingual or palatal surfaces. BOP was examined directly after the PD measurement and was reported as absent (0) or present [1]. 30 s after applying the periodontal probe. The proportion of teeth displaying BOP was recorded.

In the context of the 2017 World Workshop, a case is considered chronic periodontitis if the interdental CAL is detectable at 2 non-adjacent teeth or the buccal or oral CAL is 3 mm with pocketing > 3 mm and is detectable at ≥ 2 teeth.

The severity of periodontitis was determined as per the 2017 Update to the 1999 Classification of Periodontitis [63]. Based on the severity and complexity of management, periodontitis was classified into four stages:

##### Stage I

initial periodontitis (CAL = 1–2 mm, PD **≤** 4 mm, horizontal bone loss of the coronal third (**15**%), no tooth loss due to periodontitis).

##### Stage II

moderate periodontitis (CAL = 3–4 mm, PD **≤** 5 mm, horizontal bone loss of the coronal third (15–33%), no tooth loss due to periodontitis).

##### Stage III

severe periodontitis + potential of additional tooth loss (CAL 5 mm, PD 6 mm, horizontal bone loss extending to the middle third or beyond, vertical bone loss 3 mm, furcation involvement class II or III, and tooth loss due to periodontitis **≤** 4).

##### Stage IV

severe periodontitis + potential loss of dentition (as stage III + tooth loss due to periodontitis ≥ 5, mobility ≥ 2, need for complex rehabilitation).

#### Assessment of bone mineral density

Dual-energy X-ray absorptiometry (DEXA scan) is an advanced technology that could detect bone mineral density. The GE Lunar Prodigy densitometer was used to perform this test. To determine what constitutes a healthy bone, we utilized the World Health Organization’s criteria: a T score of -1 indicates normal bone, a T score between − 1 and − 2.5 indicates osteopenia, and a T score of -2.5 indicates osteoporosis.

#### Measurement of serum prolactin level

Blood was withdrawn from all patients to determine the fasting serum prolactin level. Prolactin concentrations in blood were measured in a faculty laboratory by automated immunoassay methodology [64].

### Data and sources of bias management

To reduce potential bias, the second investigator (psychiatrist) was blinded, as he was unaware of the patient’s oral condition and all steps after referral of psychiatric records. Periodontal assessment and enrollment were carried out by the third investigator (M.C.) who was unaware of the details of the patient’s psychological status and records. The primary investigator (S.R.) was also blinded to the steps of clinical evaluation. She collected all patient records to perform the final analysis and statistics.

### Statistical methods

Quantitative (continuous) variables were expressed as mean ± SD. As the quantitative variables were not normally distributed, the Kruskal-Wallis test (a non-parametric test) was used to compare them among the three study groups. A post hoc analysis was done for variables that showed a statistically significant difference in the Kruskal-Wallis test using Dwass-Steel-Critchlow-Fligner pairwise comparisons. As for qualitative variables (categorical), the chi-square test was used to compare them among the 3 study groups, and values were expressed as percentages. Also, linear regression analysis was performed to study the relation between two quantitative variables (serum prolactin level versus CAL and PD measurements) considering potential confounders such as age, gender, and smoking habit, while ordinal logistic regression was used to relate one quantitative (serum prolactin level) and one qualitative (BMD or stages of periodontitis) variable.

## Results

The 64 participants consisted of 21 in Group A (schizophrenic patients taking prolactin-raising antipsychotics), 21 in Group B (schizophrenic patients taking prolactin-sparing antipsychotics), and 22 in Group C (newly diagnosed schizophrenic patients not taking antipsychotics). The 21 in group A were 14 females and 7 males with a mean age of 48 ± 86.77, while the 21 in group B were 9 females and 12 males with a mean age of 47 ± 77.94, and the 22 in group C were 12 females and 10 males with a mean age of 45 ± 88.85. There was no statistically significant difference between the studied groups regarding age, gender, or smoking habits, as shown in Table 1.

**Table 1:** Characteristics of the study participants

		Group A (n = 21)	Group B (n = 21)	Group C (n = 22)	Total	P-value
Gender	Female	13(30.2%)	14(32.6%)	16(37.2%)	42(67.2%)	0.807
	Male	8(38.1%)	7(33.3%)	6(28.6%)	21(32.8%)	
Age	≤ 50 years	7(26.9%)	12(46.2%)	7(26.9%)	26(40.6%)	0.170
	≥ 50 years	14(36.8%)	6(23.7%)	15(39.5%)	38(59.4%)	
Smoking	Non-smokers	5(29.4%)	7(41.2%)	5(29.4%)	17(26.6%)	0.69
	Smokers	16(34%)	14(29.8%)	17(36.2%)	47(73.4%)	

Chi-square test

Results of the Kruskal-Wallis test revealed a statistically significant difference in PD, CAL, and serum prolactin level among the 3 study groups (P ≤ 0.001, P = 0.001, and P ≤ 0.001 respectively), while BOP was slightly significant (P = 0.058), whereas mobility was not significantly different among the 3 groups (P = 0.214), as shown in Table 2.

**Table 2:** Comparison of periodontal and serum prolactin measurements in the three study groups

Variables	Participants	N	Mean ± SD	SE	95% Confidence Interval		Kruskal-Wallis (non-parametric)		
					Lower	Upper	χ²	P-value	ε²
Pocket depth (mm)	Group A	21	5.38 ± 1.47	0.320	4.71	6.05	16.74	< 0.001	0.2658
	Group B	21	4.00 ± 1.7	0.372	3.22	4.78			
	Group C	22	3.18 ± 1.68	0.358	2.44	3.93			
CAL (mm)	Group A	21	5.81 ± 2.48	0.542	4.68	6.94	13.13	0.001	0.2084
	Group B	21	3.95 ± 2.27	0.495	2.92	4.99			
	Group C	22	2.77 ± 2.64	0.562	1.60	3.94			
BOP (%)	Group A	21	49.27 ± 26.15	5.707	37.36	61.17	5.68	0.058	0.0902
	Group B	21	47.80 ± 25.66	5.599	36.12	59.48			
	Group C	22	32.04 ± 23.9	5.100	21.43	42.64			
Mobility (%)	Group A	21	11.53 ± 8.81	1.922	7.52	15.54	2.85	0.240	0.0453
	Group B	21	14.93 ± 14.29	3.118	8.42	21.43			
	Group C	22	22.08 ± 21.99	4.687	12.33	31.83			
Serum prolactin level(ng/ml)	Group A	21	85.87 ± 55.35	12.078	60.68	111.07	30.56	< 0.001	0.4851
	Group B	21	33.64 ± 33.17	7.239	18.54	48.74			
	Group C	22	13.52 ± 4.13	0.881	11.69	15.36			

**Fig. 1:**
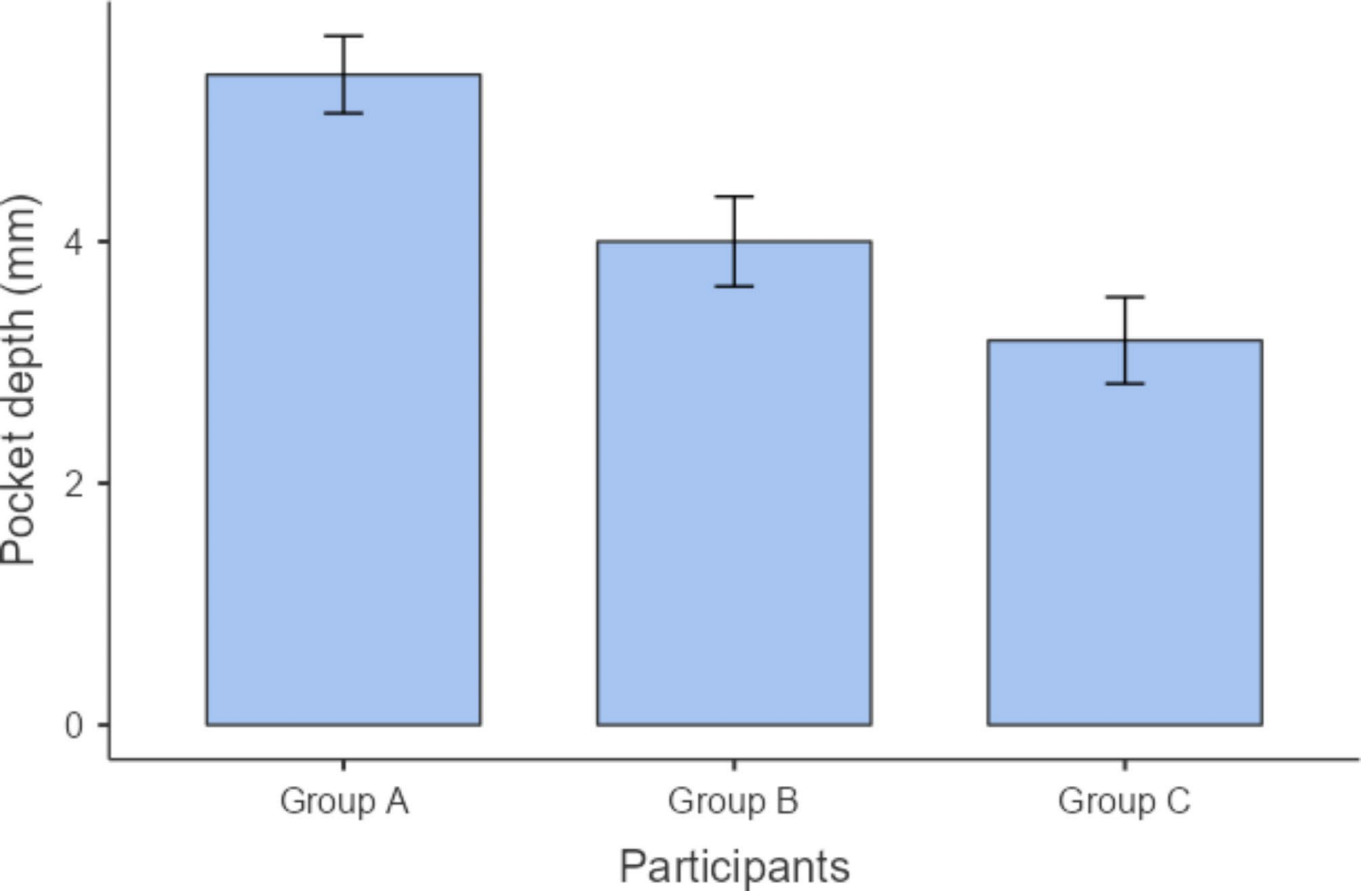
Pocket depth (mm) measurements among the 3 study groups

**Fig. 2:**
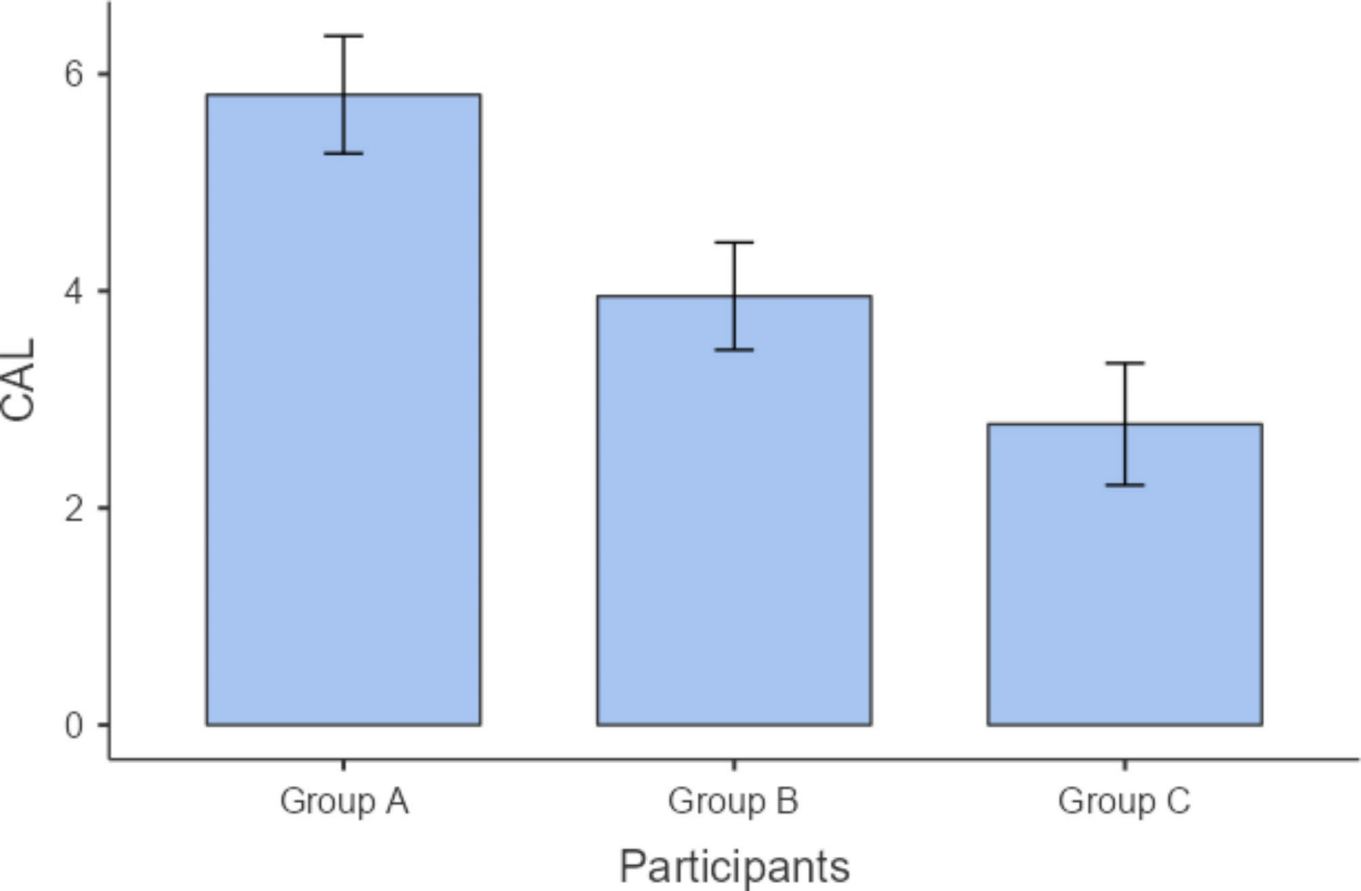
CAL (mm) measurements among the 3 study groups

**Fig. 3:**
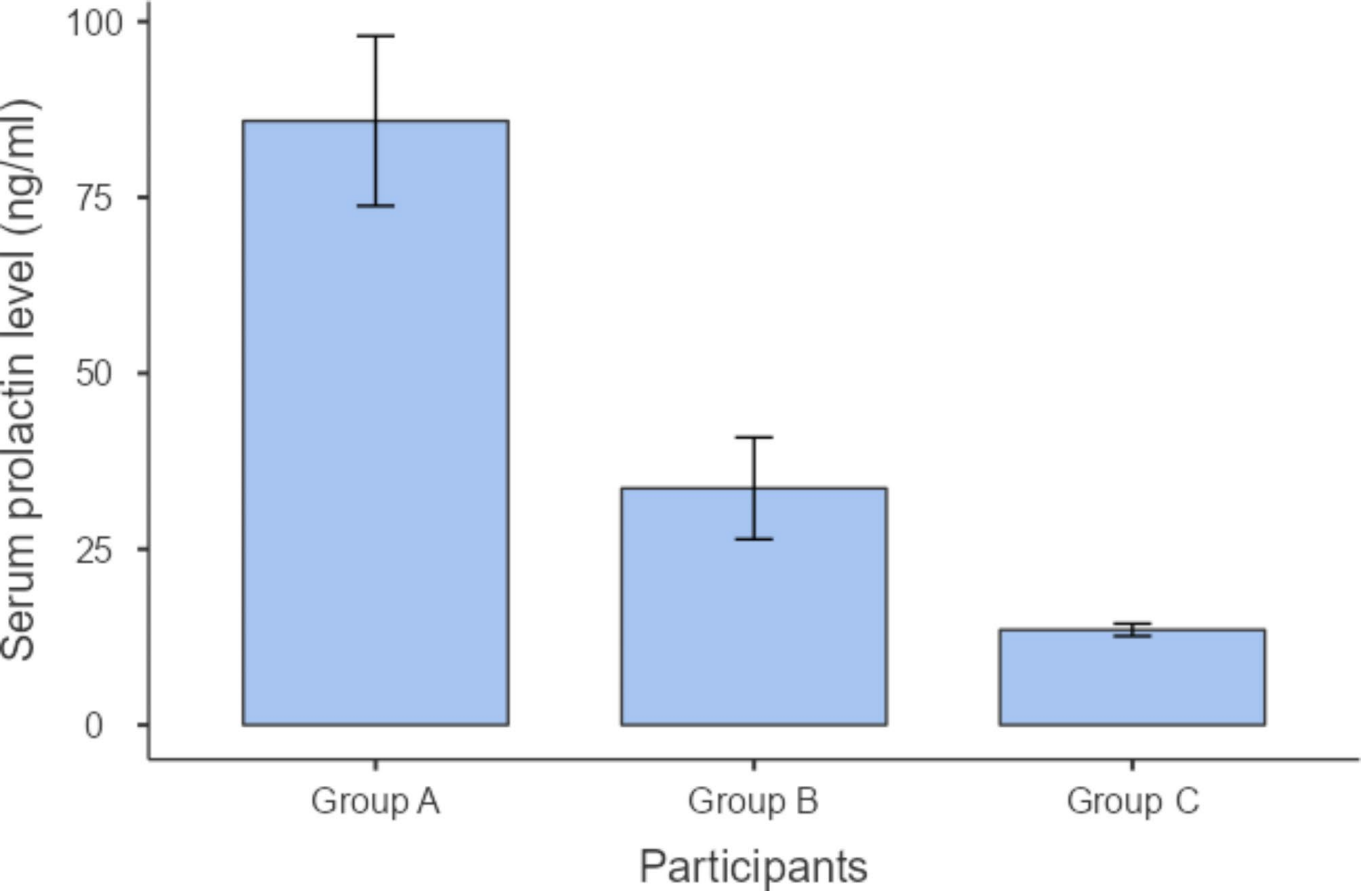
Serum prolactin level (ng/ml) among the 3 study groups

**Fig. 4:**
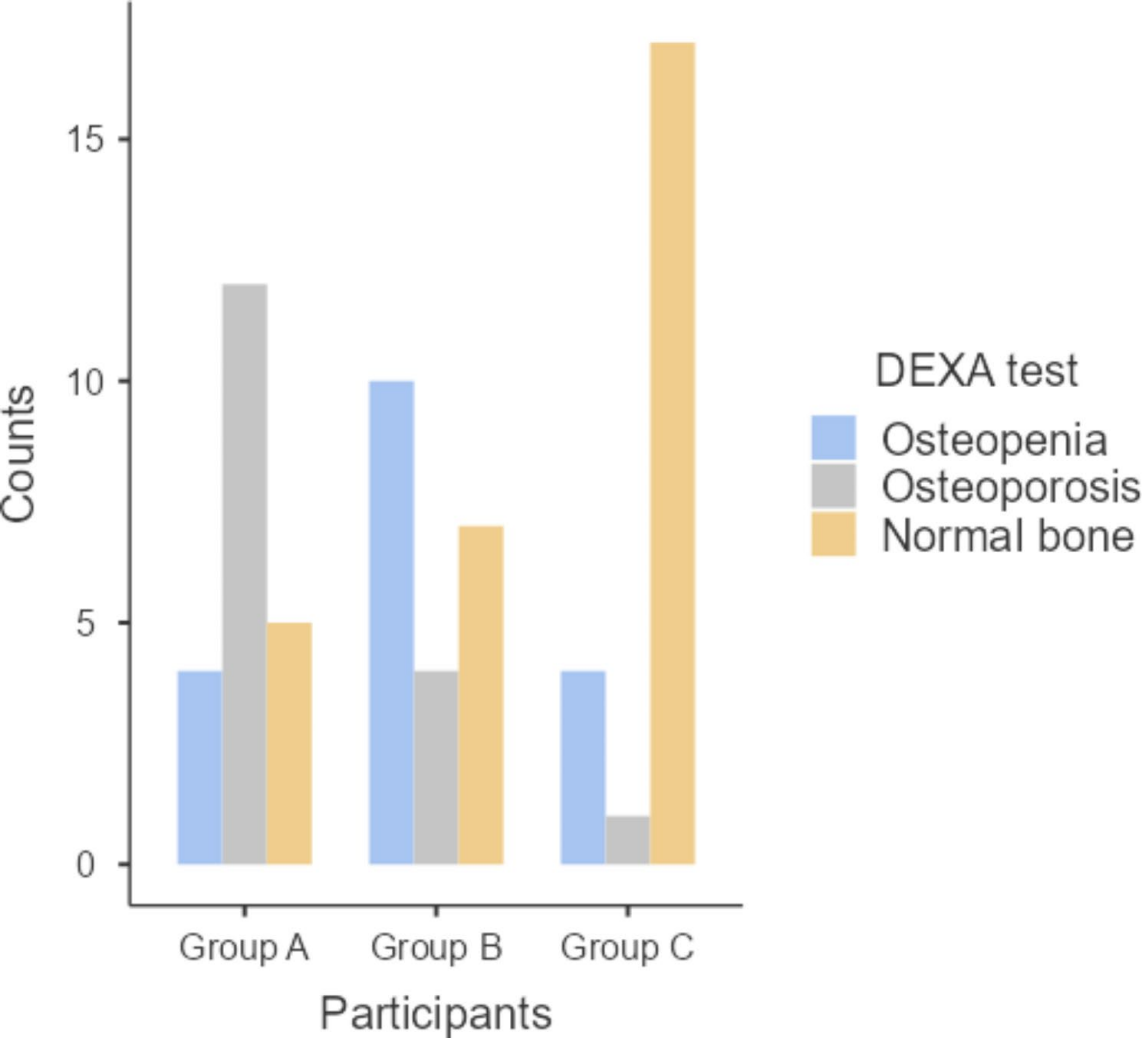
Bone mineral density (BMD) among the 3 study groups

We performed post hoc analysis for the variables that have shown statistically significant differences, as demonstrated in Table 3. Regarding PD measurements, group A has shown significantly higher values than both groups B and C (P = 0.022 and ≤ 0.001), whereas there was no statistically significant difference between the values of groups C and B (P = 0.241) as shown in Table 3 and Fig. 1. Similarly, CAL measurements of group A demonstrated significantly higher values than those of groups B and C (P = 0.042 and 0.003, respectively), but there was no statistically significant difference between the values of groups C and B (P = 0.207) as shown in Table 3 and Fig. 2. Concerning serum prolactin levels, group A had significantly higher values than groups B and C (P.001 and.001). Also, group B has shown statistically significant higher values than group B (P = 0.045) as shown in Table 3 and Fig. 3. As regards the DEXA test, the chi square test revealed a statistically significant difference (P ≤ 0.001) between the 3 study groups in terms of bone mineral density classified as patients having osteoporosis, osteopenia, or normal bone, as shown in Table 4. As shown in Fig. 4, the majority of patients in group A (57.1%) had osteoporosis, while most of the patients in group B had osteopenia (47.6%), whereas normal bone was most prevalent among patients in group C (77.3%).

**Table 3:** Post hoc analysis of the pocket depth, CAL and serum prolactin level in the 3 study groups

Variables	Groups	W	p
Pocket depth (mm)	Group A x Group B	-3.76	0.022
	Group A x Group C	-5.53	< 0.001
	Group B X Group C	-2.28	0.241
CAL (mm)	Group A x Group B	-3.41	0.042
	Group A x Group C	-4.70	0.003
	Group B X Group C	-2.40	0.207
Serum prolactin level (ng/ml)	Group A x Group B	-5.37	< 0.001
	Group A x Group C	-7.18	< 0.001
	Group B X Group C	-3.37	0.045

Dwass-Steel-Critchlow-Fligner pairwise comparisons

**Table 4:** Comparison of DEXA test and severity of periodontitis in the three study groups

Variable	N	χ²			Effect size	Standard Error	95% Confidence Intervals	
		Value	df	p	Gamma		Lower	Upper
DEXA test	64	28.21	8	< 0.001	0.378	0.131	0.121	0.636
Severity of Periodontitis	64	23.6	6	< 0.001	0.256	0.146	-0.029	0.541

Chi-square tests Note. The variable ‘Severity of periodontitis’ has the following order: Stage I | Stage II | Stage III | Stage IV and the variable ‘DEXA test’ has the following order: Osteopenia | Osteoporosis | Normal bone

**Fig. 5:**
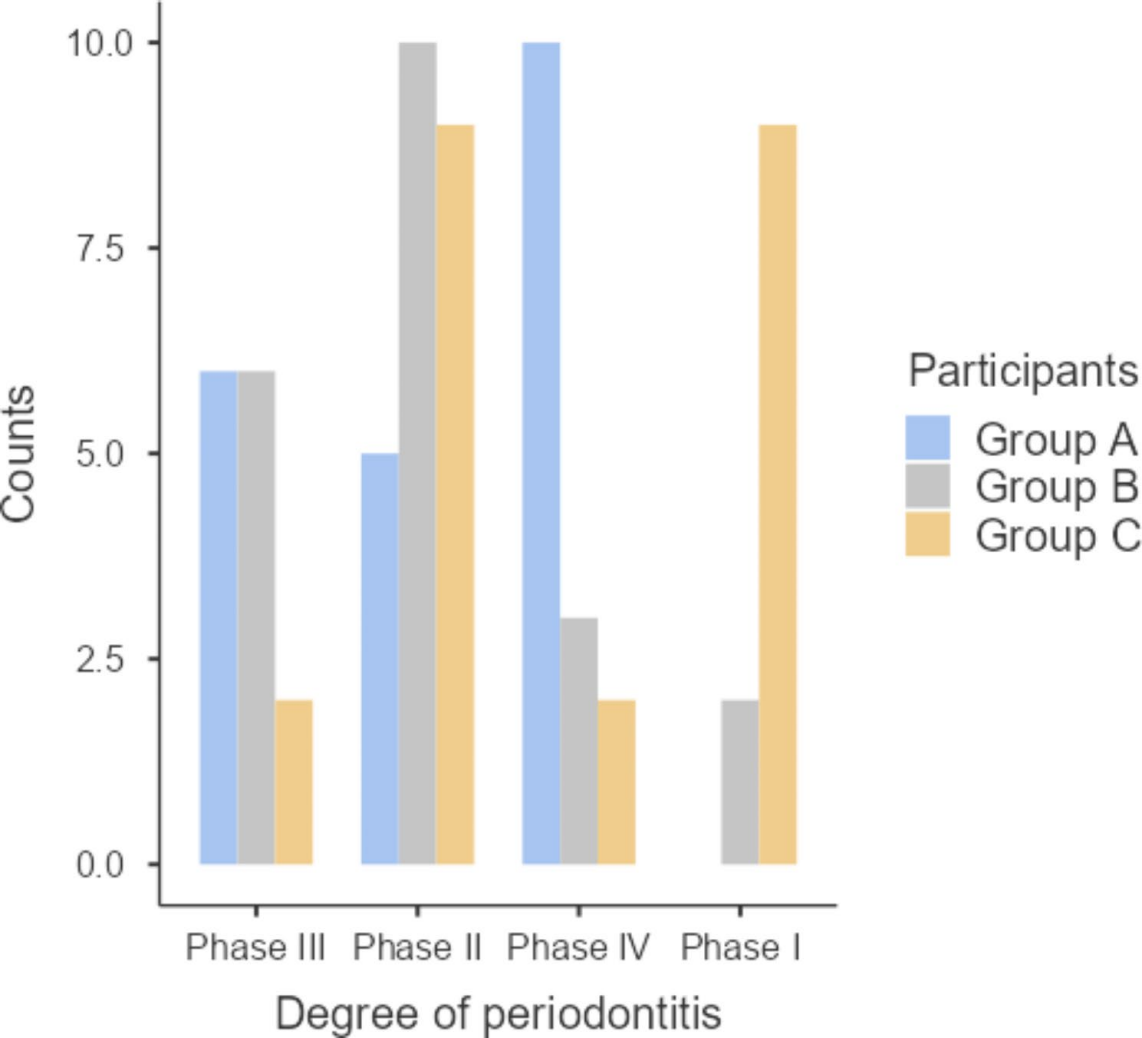
Severity of periodontitis among the 3 study groups

Moreover, by comparing the distribution of different stages of periodontitis, whether Stage I, Stage II, Stage III, or Stage IV periodontitis, results yielded a statistically significant difference among the 3 study groups (P ≤ 0.001). As shown in Fig. 5 and Table 4, the majority of patients in group A had stage IV periodontitis (47.6%). However, most of group B had stage II periodontitis (47.6%).

**Table 5:** Linear regression analysis between Serum prolactin level (predictor) versus Pocket depth and CAL (outcome)

Model Fit Measures			Overall Model Test				
Serum prolactin level (ng/ml) (Predctor) and CAL (mm) (outcome)							
R		R²	Predictor	Estimate	SE	t	p
0.430		0.185	Intercept	3.3583	0.775	4.33	< 0.001*
Model Coefficients – CAL (mm)			Serum prolactin level (ng/ml)	0.0217	0.00684	3.169	0.002*
Gender				0.757	0.69	1.094	0.279
Age				-0.342	0.658	-0.521	0.605
Smoking				-0.387	0.733	-0.528	0.600
**Serum prolactin level (ng/ml) (Predctor) and Pocket depth (mm) (outcome)**							
**R**	**R²**		**Predictor**	**Estimate**	**SE**	**t**	**p**
**0.500**	**0.250**		Intercept	3.823	0.502	7.606	< 0.001*
**Model Coefficients - Pocket depth (mm)**			**Serum prolactin level (ng/ml)**	**0.015**	0.0044	3.383	0.001*
**Gender**				**-0.395**	0.426	-0.926	0.359
**Age (≥ or ≤ 50y)**				**0.876**	0.449	1.95	0.056
**Smoking**				**-0.5627**	0.475	-1.182	0.242

*Statistically significant

Upon performing regression analysis, which could reflect the possible causal correlation between serum prolactin level (ng/ml) (the predictor) and CAL as well as PD measurements (mm) (the outcome), results yielded a statistically significant direct relation (P = 0.002, 0.001, respectively), which indicates that an increase in serum prolactin level could possibly cause an increase in CAL and PD measurements, which are the main indicator for diagnosis and severity of periodontitis, as well as PD measurements, as shown in Table 5. In this regression relation, the R2 was adjusted for the possible confounders (age, sex, and smoking habit). Furthermore, we performed logistic regression to test the assumption of serum prolactin level as a risk factor (predictor) for both the bone mineral density (measured by DEXA test) as well as the severity (stages) of periodontitis (outcomes), and the results revealed a statistically significant direct relation (with odds ratios of 0.991 and 0.988, respectively), as shown in Table 6.

**Table 6:** Ordinal logistic regression analysis between Serum prolactin level (as predictor) in relation to severity of periodontitis and bone mineral density (DEXA test) (as outcomes)

Model Fit Measures					Model Coefficients						
		Overall Model Test								95% Confidence Intervals	
Outcome	R²_N_	χ²	df	p	Estimate	SE	Z	p	Odds ratio	Lower	Upper
Severity of periodontitis	**0.0453**	**5.74**	**1**	**0.017**	-0.0124	0.00542	-2.28	0.022	0.988	0.976	0.998
DEXA test	0.0362	3.57	1	0.059	-0.00918	0.00502	-1.83	0.067	0.991	0.981	1

Note: The variable ‘Severity of periodontitis’ has the following order: Stage I | Stage II | Stage III | Stage IV. The variable ‘DEXA test’ has the following order: Osteopenia | Osteoporosis | Normal bone

## Discussion

Schizophrenia is one of the most common causes of disability worldwide. It results in a reduction in life expectancy by approximately 12–15 years less than normal. This is due to a higher suicide rate, obesity, a lack of activity, and smoking, with a higher burden of human and economic costs. It typically affects young men, aged between 20 and 28 years, about 1.4 times more than women [65].

Several hypotheses have been implicated in the pathogenesis of schizophrenia, the most acceptable of which is the dopamine hypothesis. According to this theory, psychosis is caused by a misinterpretation of the abnormal activity of dopaminergic neurons in the brain. Some studies have linked the altered dopamine metabolism with systemic infection and inflammation, which involve the interplay of cytokines causing symptoms of schizophrenia [66–70].

Of interest, a bidirectional relationship between periodontal diseases and schizophrenia has been suggested. This could be justified in one way by the fact that schizophrenia patients neglect oral hygiene in addition to the xerostomia induced by antipsychotic medication. On the other hand, schizophrenia has been associated with increased serum levels of cytokines such as IL-1R antagonists, IL-2, IL-6, and acute-phase proteins. Bearing in mind that there is upregulation of these pro-inflammatory cytokines in periodontitis, an elevation could suppress the release of glutamate and enhance dopamine survival, resulting in inhibition of N-Methyl-D-Aspartate (NMDA) glutamate receptors, which contribute to the development of schizophrenia.

Furthermore, a positive link has been found between the severity of schizophrenia and the increased prevalence of periodontal pathogens such as P. gingivalis [45, 65, 71–74].

Accordingly, a deep understanding of the possible association between periodontal disease and schizophrenia could pave the way for proper prevention and management of both diseases. However, little is known about the role of antipsychotics in the progression of periodontal diseases. Previous reports have focused on the role of xerostomia induced by antipsychotics in the progression of periodontitis, where an increase in periodontal measurements has been reported. Moreover, the possible association between periodontitis and schizophrenia has been linked to antipsychotic-induced osteoporosis [27].

Despite the fact that both serum prolactin levels and bone regulation involve complex mechanisms, hyperprolactinemia induced by antipsychotics has been implicated in bone loss. Also, regulation of serum prolactin levels is principally affected by dopamine acting on D2 receptors on lactotropes of the anterior pituitary gland, resulting in inhibition of prolactin secretion. Knowingly, antipsychotics block D2 receptors and thus increase prolactin secretion, resulting in hyperprolactinemia [44].

Concerning the role of antipsychotic-induced hyperprolactinemia in bone metabolism, two pathways have been explained. The first is the direct effect of prolactin on the induction of osteoclastic bone resorption, which is achieved by enhancing transcription of mRNA for RANKL and promoting RANK-RANKL interaction, causing osteoclast activation and thus bone resorption. The other pathway includes the endocrine regulation of prolactin through gonadotrophin-releasing hormone, which induces hypogonadism. Nevertheless, some authors claimed that there is a positive association between osteoporosis and periodontal diseases; however, it cannot be regarded as a causative factor [74, 75].

Accordingly, the present study was employed to investigate the role of antipsychotic-induced hyperprolactinemia as a risk factor for the progression of periodontitis. Current evidence on the effect of antipsychotic medication on periodontitis is limited. Our study is among the few reports that have analyzed the possible link between antipsychotics and periodontitis in schizophrenic patients [27, 29, 76–78].

To our knowledge, this is the first study to compare the effect of antipsychotics in schizophrenic patients according to their “prolactin raising” or “prolactin sparing” effect in terms of the progression of periodontal diseases. The only available study that addressed this relationship was a case report of a young woman in reproductive age who has been treated with two atypical antipsychotics, namely, risperidone and amisulpride, simultaneously. Although this patient reported a significant increase in serum prolactin level, bone demineralization, and a severe form of periodontitis, suggesting a possible correlation between these findings, these observations cannot be interpreted with certainty, not only because this is a case report but also because this is a young female with a possible hormonal disturbance [75].

Another recent animal study suggested the role of two atypical antipsychotics (Olanzapine and Clozapine) in increased bone loss and eventually periodontitis. They concluded that atypical antipsychotics could be a risk factor for periodontitis due to the induction of an inflammatory response that is irrelevant to their metabolic effect [79].

In the present study, results showed clearly that PD and CAL, as well as serum prolactin levels, were higher in the group taking prolactin-inducing antipsychotics than in the group taking prolactin-sparing antipsychotics. Additionally, these measurements were elevated in those taking antipsychotics more generally than in newly diagnosed schizophrenic patients who had not received any medication yet. Conversely, there were no significant differences in BOP or mobility among the study groups. However, considering CAL and PD measurements is more reliable, as they are the main periodontal parameters in the diagnosis and prognosis of periodontitis [63].

Furthermore, our results demonstrated the tight association between antipsychotic-induced hyperprolactinemia and reduced BMD (measured by the DEXA test) as well as the severity of periodontitis. Results revealed that most patients taking prolactin-inducing antipsychotics had osteoporosis and stage IV (severe) periodontitis, while in those taking prolactin-sparing antipsychotics, osteopenia and stage III (less severe) periodontitis were most prevalent, whereas the majority of newly diagnosed patients had normal bone with stage I or II (initial or moderate) periodontitis. We further confirmed these results by performing regression analysis to verify the direct causal relation between serum prolactin level and PD, BMD, and degree of periodontitis.

In the context of the relation between antipsychotics and periodontal diseases in schizophrenic patients, a study conducted by **Etlas et al.** [27] investigated the effect of antipsychotics on PD, CAL, BOP, and plaque index (PI), with conflicting results showing a significant association between elevation of PI and BOP and a reduction in salivary flow rate (SFR), while there was no significant correlation between PD and CAL and a reduction in SFR. Unfortunately, their results cannot be compared to ours, as they mainly classified their groups based on the effect of antipsychotics on SFR. Similar results have been found in the study conducted by **Djamaluddin et al.** [28], who concluded that periodontal diseases in schizophrenic patients are caused by reduced salivary secretion induced by antipsychotic drugs and are directly related to the duration of use of these drugs.

Despite the lack of sufficient data from the literature, a few reports confirmed the positive relationship between osteoporosis and periodontitis, which is consistent with our study. On the contrary, some other reports did not find this association between osteoporosis and periodontitis [52, 74, 80–92].

Regarding the role of antipsychotics in bone remodeling or loss, some studies recently attributed this relation to alteration of regulatory cytokines with changes in serum levels of TNF-, INF-, IL-6, IL-10, IL-17, IL-27, and TGF- in schizophrenic patients with resultant reductions in BMD and eventually osteoporosis [93–96]. Nevertheless, one confusing finding in the present study is that the majority of the prolactin-inducing antipsychotics group were females above 50 years of age, which infers some doubt in the assumption that the prevalence of osteoporosis among members of this group is attributed to hyperprolactinemia due to the well-established relation between structural bone changes and estrogen deficiency, especially in postmenopausal women [97–99].

The current available evidence from previous studies indicates that all antipsychotic medications could contribute to the acceleration and exacerbation of periodontal diseases, with a higher incidence associated with antipsychotics that induce hyposalivation. Furthermore, it has been suggested that FGAs that induce hypersalivation could possess a protective effect on periodontal tissues [29].

In conclusion, our results showed that the induction of hyperprolactinemia by antipsychotic medication was a prime factor in the progression of periodontal diseases. Accordingly, clinicians should consider avoiding this risk factor during the pharmacologic management of schizophrenic patients. This could be achieved by selecting an antipsychotic with the fewest adverse effects and avoiding the prescription of multiple pharmacologic agents whenever possible.

### Strengths, limitations, and future perspectives

The major strength of the current study is that it addressed an important topic that has been overlooked in previous literature. It deals with two common and highly prevalent health problems: psychiatric and periodontal diseases. Moreover, it is the first study to compare the effect of antipsychotic medications on periodontal disease in terms of their effect on serum prolactin levels. Despite of that schizophrenia is more common in males, the results of our study could be of special concern to female patients, who are generally more susceptible to both psychiatric problems and osteoporosis.

Secondly, we assume that the sample size was adequate, as statistical sample size calculations were performed based on previous research to avoid the unnecessary inclusion of patients. Furthermore, every effort was done to avoid potential bias ( as previously described in methods).

Another point of strength is that our study could statistically prove the causal correlation between serum prolactin level and periodontal measurements such as CAL and PD through regression analysis that excluded some important confounders, which are age, gender, and smoking habits. Although matching was not performed, however, statistical analysis yielded no significant differences between the study groups regarding these parameters.

Finally, our research could provide some guidance for psychiatrists in the prescription of antipsychotic medications, considering their effect on oral and periodontal health, as well as dentists dealing with psychiatric patients.

Limitations of the current study include the inherent weakness of retrospective studies that depend on patients’ databases and prescription claim records, which did not enable us to properly evaluate patients’ compliance with treatment. Additionally, although eligibility criteria involved systemically free patients, as systemic medical conditions could possibly act as confounders, it was difficult to guarantee their absence due to reduced patient cooperation during history-taking as well as inaccuracies in some records. Accordingly, we anticipate that some factors might be overlooked. The non-availability of enough information about potential risk factors such as dietary habits and lifestyle is one considerable limitation of the current study.

Another point of weakness is that comparisons were performed according to drug category, ignoring differences that might exist between individual types of drugs and different dosing regimens. However, we tried to focus on the main objective of our study, which is comparing antipsychotics based on their effect on serum prolactin level. Finally, one important consideration is the lack of knowledge about patients’ oral and periodontal conditions before receiving antipsychotic medication. Therefore, we tried to avoid this shortcoming by considering a third group of “newly diagnosed patients” as a baseline point for comparison.

Furthermore, long-term interventional and controlled studies are required to increase our understanding of the impact of antipsychotic-induced hyperprolactinemia in relation to periodontal diseases. Additionally, both researchers and practitioners should collaborate to increase awareness about the effects of antipsychotic-induced hyperprolactinemia on physical and oral health. For this reason, integration between regular dental and periodontal evaluation and screening of serum prolactin levels as well as BMD in phychotic management is recommended. A preventive dental program should be increased for this vulnerable group of patients.

## Data Availability

The data used to support the findings of this study are included in this published article.
